# Desvenlafaxine As the Main Possible Culprit in Triggering Reversible Cerebral Vasoconstriction Syndrome: A Case Report

**DOI:** 10.7759/cureus.29780

**Published:** 2022-09-30

**Authors:** Mohammad Abu-Abaa, Malik AbuBakar, Aliaa Mousa, Daniel Landau

**Affiliations:** 1 Internal Medicine, Capital Health Regional Medical Center, Trenton, USA; 2 Neurology, Capital Health Regional Medical Center, Trenton, USA

**Keywords:** thunder clap headache, stroke-like symptoms, serotonin-norepinephrine reuptake inhibitors, reversible cerebral vasoconstriction syndrome (rcvs), desvenlafaxine

## Abstract

Reversible cerebral vasoconstriction syndrome (RCVS) is not an uncommon condition. It should be suspected in young patients with new onset headaches and neurologic deficits. We report a 38-year-old male patient with a history of depression on desvenlafaxine for two years and no other triggering factor who was diagnosed with RCVS confirmed by cerebral angiogram. Discontinuation of the medication and calcium channel blockers initiation led to rapid clinical improvement. The diagnosis was further confirmed by angiographic improvement two months later. Although the association of selective serotonin reuptake inhibitors (SSRI)/ serotonin norepinephrine reuptake inhibitors (SNRI) with RCVS has been reported frequently, desvenlafaxine is a much less reported trigger, with only nine cases in total. In contrast to prior reported cases where the time from exposure to onset of RCVS was weeks to months, the time interval, in this case, was two years. This case report aims to support previous literature in suggestion of this association.

## Introduction

Reversible cerebral vasoconstriction syndrome (RCVS) is a clinical and radiological syndrome with unknown true incidence. RCVS remains an underdiagnosed condition that usually presents with a severe thunderclap headache with possible focal neurological deficits and subarachnoid hemorrhage. Cerebral angiography confirms the diagnosis by demonstrating focal areas of stenosis and diltation. Diagnosis also requires confirmation of recovery of intracranial stenosis seen in cerebral angiogram a few months later. It is usually a medication-induced condition in 25-60% of cases, likely by alteration of cerebral vascular tone. The most commonly implicated medications include sympathomimetics, anti-migrainous, and anti-depressant medications, especially selective serotonin reuptake inhibitors (SSRI)/ serotonin-norepinephrine reuptake inhibitors (SNRI). Association with postpartum state, blood pressure surges, and migraine has been well documented. It can also happen spontaneously. Endothelial dysfunction is likely given the overlap with posterior reversible encephalopathy syndrome (PRES). It usually affects those between 20-50 years of age, though it also can affect children and adolescents as well. The female-to-male ratio is 2.4:1, and females tend to be affected on average a decade later than men [1]. This article is aimed to support prior reports of the association between RCVS and desvenlafaxine. 

## Case presentation

**Figure 1:**
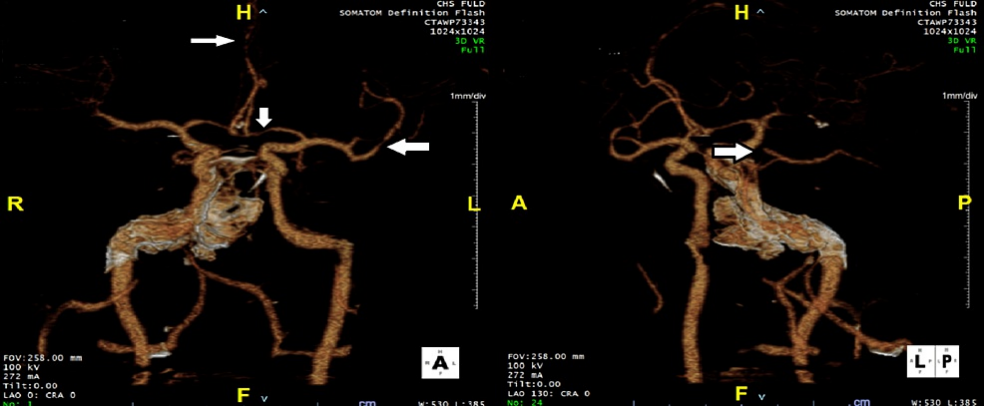
Initial computed tomography angiography (CTA) of the brain CTA brain showing multifocal stenosis affection left M2, left A1, bilateral A3, and right P1 (arrows).

**Figure 2:**
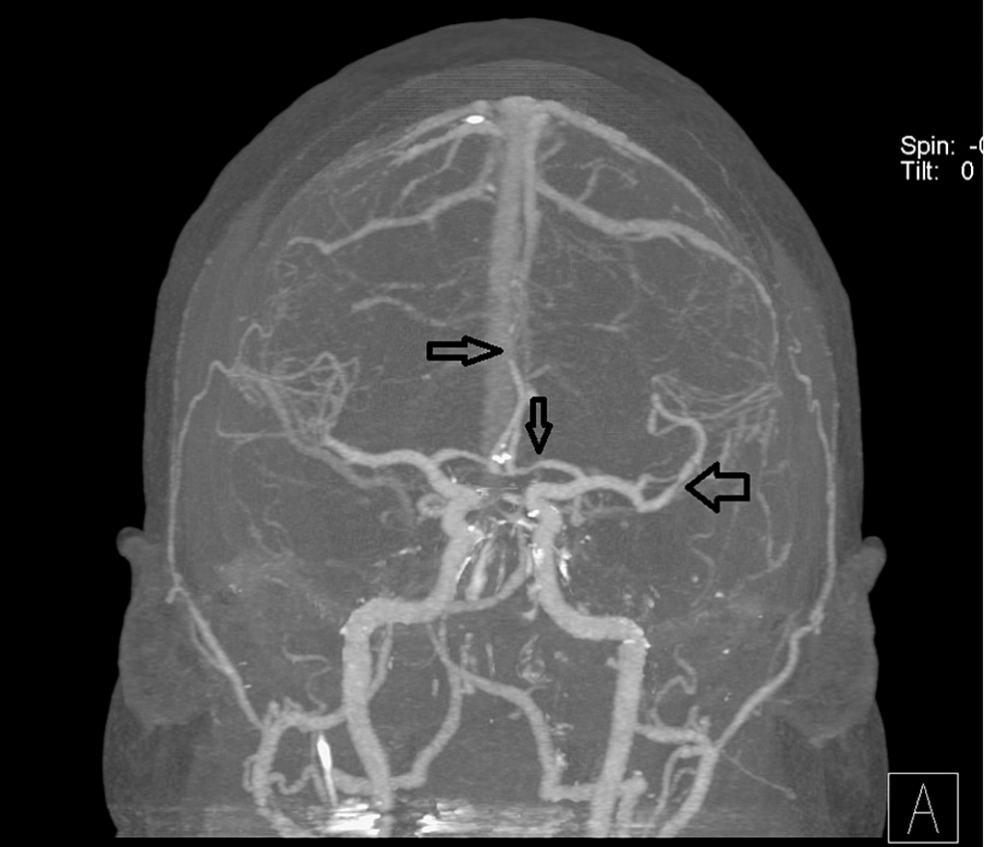
Initial computed tomography angiography (CTA) of the brain CTA showing multifocal stenosis affecting left M2, A1, and bilateral A3 (arrows).

A 38-year-old male patient presented with a three-day history of gradual onset vertigo with bilateral frontal and occipital pulsating headache, right-sided facial numbness, and left visual impairment. He also described a transient episode of slurred speech and difficulty walking. Past medical history is significant for type 1 diabetes on insulin and depression. He was on desvenlafaxine 100 milligrams daily for the last two years. No other medications were in use, including over-the-counter medications. Social history was also unremarkable for alcohol, marijuana, and illicit drug use. Physical exam showed left lower temporal quadrantanopia, and diminished light touch sensation over the right lower face over V1 to V3 distribution, and otherwise was unremarkable. Vital signs were stable. Basic lab work was unremarkable, including a toxicology screen. Computed tomography (CT) scan of the head was unremarkable, with no evidence of subarachnoid hemorrhage. However, CT angiography (CTA) of the head showed multifocal stenosis involving left inferior M2, left A1, bilateral A3, and right P1 (Figure 1, 2). This was confirmed by a cerebral angiogram. No extracranial stenosis or dissection was seen. Magnetic resonance imaging (MRI) of the brain was unremarkable, with no evidence of posterior reversible encephalopathy syndrome (PRES). Lumbar puncture (LP) was unyielding. This raised suspicion of reversible cerebral vasoconstriction syndrome. The patient was managed with verapamil. 

**Figure 3:**
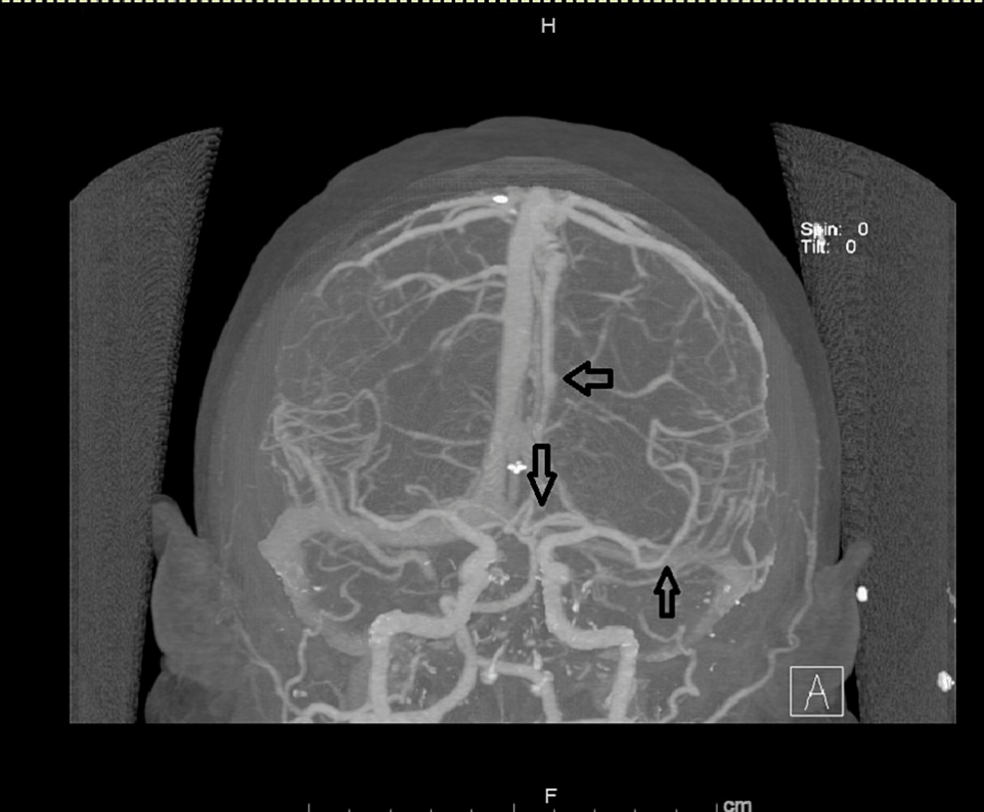
A follow-up computed tomography angiography (CTA) of the brain CTA brain showing relative improvement of prior multifocal stenosis affecting left M2, left A1, and A3 (arrows).

Extensive workup for vasculitis, including antinuclear antibody (ANA), cytoplasmic antineutrophil cytoplasmic antibody (C-ANCA), perinuclear antineutrophil cytoplasmic antibody (P-ANCA), anti-Smith, anti-dsDNA, anti-Ro/La, anti-RNP, antiphospholipid antibodies, complement, and angiotensin-converting enzyme (ACE) levels were unyielding. Clinical response to verapamil was appreciated rapidly, and his neurological exam normalized on the second day of hospitalization. Depression management was changed to mirtazapine. All symptoms have resolved at the time of discharge from the hospital. CTA brain was repeated two months later with evidence of resolution of multifocal cerebral stenosis (Figure 3).

## Discussion

RCVS is not a single disease entity but rather a common presentation of various disorders characterized by cerebral vasoconstriction, including cell-fleming syndrome, a thunderclap headache, and postpartum angiopathy. The key radiographic feature is the presence of segmental arterial vasoconstriction, which may be absent early [1]. Although RCVS can happen spontaneously, a trigger can be identified in 25-60% of cases, and in 50-60% of cases, it is related to the postpartum state [2]. Most of the currently available evidence on the association of RCVS with SSRI/SNRI is based on case reports and case series. Cases have been reported in association with venlafaxine, duloxetine, paroxetine, citalopram, fluoxetine, and sertraline [3]. There is a smaller body of knowledge regarding the association of RCVS with desvenlafaxine. It is mainly based on case reports [4]. A review of drug adverse events data from the Food and Drug Administration (FDA) shows only nine reported cases of desvenlafaxine-associated RCVS. 

Naranjo score is used to calculate the probability of a drug-induced adverse event. Given the occurrence of RCVS while on desvenlafaxine, confirmed finding by angiogram, improvement after its discontinuation, and lack of other identifiable previously reported triggers, the calculated Naranjo score is at six. This indicates the probability of desvenlafaxine-induced RCVS [5].

It is very difficult to differentiate between RCVS and primary angiitis of the central nervous system (PACNS). A better approach to spare a young age patient with severe a headache and neurological deficits from immunosuppressants is to try calcium channel blockers. Those with RCVS are expected to improve rapidly. It should be noted that steroids may worsen RCVS [3,6].

Therefore, it is important to differentiate between the two entities. All patients with PACNS have abnormal brain CT/MRI, while those with RCVS can have initially normal imaging in 20% of cases. CSF analysis is usually unremarkable in RCVS, while those with PACNS usually have lymphocytic pleocytosis. Multiple deep lacunes, deep white matter lesions, tumor-like lesions, and multiple gadolinium-enhancing lesions are seen only in PACNS, while carotid artery dissection is seen only in RCVS. Angiographic abnormalities happen in both [7].

There is no randomized clinical trial to provide guiding evidence in the management of RCVS. Discontinuation of a triggering agent is mandatory. The most commonly used medications include verapamil, nicardipine, and nimodipine, all of which are based on case series. The two case series in France and Taiwan had consistent findings regarding the efficacy of calcium channel blockers (CCB) in improving the outcomes [8,9]. However, the largest case series in the USA showed no benefit of nimodipine over symptomatic therapy alone [3]. In our case, rapid clinical response was achieved with verapamil.

## Conclusions

A typical presentation of reversible cerebral vasoconstriction syndrome (RCVS) is a severe headache with or without neurological deficits and subarachnoid hemorrhage. Numerous medications have been implicated in triggering RCVS and in such case discontinuation of the inciting factor can lead to resolution of RCVS. Desvenlafaxine is less commonly reported as an inciting factor of RCVS as compared to other SNRI/SSRI. The interval between exposure to medication and onset of symptoms can be years as shown by this case. In majority of cases, discontinuation of the inciting medication leads to resolution of the symptoms. 
